# Corneal epithelial aberrations: a novel diagnostic tool for keratoconus and forme fruste keratoconus

**DOI:** 10.1186/s40662-025-00449-x

**Published:** 2025-08-06

**Authors:** Rui Ning, Chak Seng Lei, Xinning Yang, Yue Li, Yizhou Yang, Ingemar Gustafsson, Giacomo Savini, Domenico Schiano-Lomoriello, Xingtao Zhou, Xiaoying Wang, Jinhai Huang

**Affiliations:** 1 Eye Institute and Department of Ophthalmology, NHC Key Laboratory of Myopia and Related Eye Diseases; Key Laboratory of Myopia and Related Eye Diseases, Eye & ENT Hospital, Fudan University, Chinese Academy of Medical Sciences, Shanghai, China; 2 Shanghai Research Center of Ophthalmology and Optometry, Shanghai, China; 3 School of Health Science and Engineering, University of Shanghai for Science and Technology, Shanghai, China; 4 Eye Hospital and School of Ophthalmology and Optometry, Wenzhou Medical University, Wenzhou, Zhejiang China; 5 Department of Clinical Sciences, Ophthalmology, Lund University, Skåne University Hospital, Lund, Sweden; 6 G.B. Bietti Foundation I.R.C.C.S., Rome, Italy

**Keywords:** Corneal epithelial aberrations, Diagnostic, Keratoconus, Forme fruste keratoconus

## Abstract

**Purpose:**

To assess the ability of corneal epithelial aberrations to discriminate forme fruste keratoconus (FFKC) and keratoconus (KC) from normal eyes.

**Methods:**

This prospective, case-control study enrolled 91 right eyes from 91 normal participants, 87 eyes with FFKC and 148 eyes with KC. Epithelial aberrations for the 6-mm pupil were measured using an anterior segment optical coherence tomography (MS-39, CSO). The epithelial root mean square of higher and lower-order aberrations (total RMS), root mean square of higher-order aberrations (HOAs RMS, from the 3rd to the 7th Zernike polynomials), coma, trefoil, spherical aberration, and secondary astigmatism were recorded. Stepwise logistic regression was utilized to develop the epithelial aberrations index (EAI) for obtaining the optimal discriminant function to diagnose FFKC (EAI-FFKC) and KC (EAI-KC). Area under the receiver operating characteristic curve (AUC) analysis was used to determine the diagnostic accuracy of the indices.

**Results:**

FFKC and KC eyes had significantly higher epithelial aberrations than normal eyes. Comparing FFKC with the normal group, epithelial HOAs RMS and coma attained AUC values of 0.714 and 0.788, respectively. The EAI-FFKC showed the highest discrimination ability to differentiate FFKC from normal eyes indicated by an AUC value of 0.822 with 77.0% sensitivity and 75.8% specificity. Comparing KC with the normal group, epithelial HOAs RMS attained AUC values of 0.976–0.998 with 95.2%–100% sensitivity and 92.3%–96.7% specificity, epithelial coma attained AUC values of 0.974–0.997 with 92.9%–100% sensitivity and 96.7%–98.9% specificity. The EAI-KC showed the highest discriminative ability to differentiate KC from normal eyes indicated by AUC of 0.996 with 98.6% sensitivity and 98.9% specificity.

**Conclusion:**

Epithelial wavefront analysis can identify abnormal epithelial changes across all stages of KC, from very early to severe. Epithelial aberrations can be used as a diagnostic tool for KC and FFKC.

**Supplementary Information:**

The online version contains supplementary material available at 10.1186/s40662-025-00449-x.

## Background

Keratoconus (KC) is a bilateral, asymmetric, progressive eye disorder characterized by the thinning and steepening of the cornea, leading to significant visual impairment, high astigmatism, corneal scarring, and a reduced quality of life [1–3]. Corneal collagen cross-linking is the most effective way to halt the progression of KC when performed early, before extensive corneal thinning and distortion occur, thereby avoiding loss of vision and preventing the need for keratoplasty [4]. Therefore, early detection method with high accuracy plays a pivotal role for effective screening and management.

In normal corneas, the corneal epithelium is relatively uniform in its thickness [5, 6], however, its pattern shifts to central thinning and peripheral thickening, usually resembling a "epithelial doughnut pattern" in keratoconic cornea [7, 8]. In the very early stage of KC, the epithelium remodeling ability will compensate the stromal irregularities to maintain a smooth anterior surface resembling a normal cornea. This epithelial compensation mechanism can be observed throughout the course of KC and the degree of compensation is dependent on the severity [7]. The corneal epithelium exhibits dynamic, metabolically active compensatory remodeling processes in response to biomechanical stress redistribution and subclinical stromal irregularities. Wavefront serve as quantitative morphological biomarkers demonstrating significant sensitivity to these epithelial adaptive mechanisms [9, 10]. Prior research has established the critical role of higher-order aberrations (HOAs) in understanding KC progression [11–13]. However, these studies focused on total HOAs without isolating the epithelial contribution, leaving a gap in understanding regarding how epithelial remodeling contributed in early KC. This mechanism can mask the detection of underlying stromal variation when using conventional topographers like Scheimpflug camera or Placido-disk, leading to a missed diagnosis [14, 15]. Instead, anterior segment optical coherence tomography (AS-OCT) allows for detailed analysis of multiple layers of the cornea and is capable of identifying minor alterations in the corneal epithelium [16]. In this study, the MS-39 AS-OCT (CSO, Florence, Italy) was used, as it can perform epithelial wavefront analysis by calculating the difference in height between the wavefront generated by the measured corneal epithelium and an ideal wavefront within the analysis diameter, and demonstrating the amounts of epithelial aberrations in Zernike polynomials. Hence, we hypothesized that this analysis could provide quantitative data for revealing early abnormal changes of the epithelium in patients with KC, which could be of clinical relevance.

To the best of our knowledge, no previous studies have analyzed corneal epithelial aberrations in KC eyes. In this prospective study, we evaluated the diagnostic potential of epithelial aberration analysis by systematically comparing measurements across three participant groups: healthy controls, forme fruste keratoconus (FFKC), and established KC at varying disease stages.

## Methods

### Participants

This prospective study was conducted with the approval of the Ethics Committee of the Eye and ENT Hospital of Fudan University (Shanghai, China) with reference no. 2021175 and adhered to the principles of the Declaration of Helsinki. All participants signed an informed consent after having received information regarding the objectives and procedures of the study.

The study enrolled patients diagnosed with KC at the Eye & ENT Hospital of Fudan University. All participants underwent a full ophthalmic examination, which consisted of best-corrected visual acuity (BCVA) using a standard Tumbling E Visual Chart, slit-lamp examination, non-contact tonometry (TX-20P, Canon, Tokyo, Japan), indirect ophthalmoscopy, corneal tomography (Pentacam, Oculus, Wetzlar, Germany) and AS-OCT (MS-39, CSO, Florence, Italy). The inclusion criteria for the KC group included abnormal tomography, with specific attention to anterior keratometry, posterior elevation, thinnest pachymetry and skewed asymmetric bowtie pattern [17]. Based on the topographical keratoconus classification (TKC) provided by Pentacam software, the KC group was subdivided into three subgroups: Mild (TKC = 1/1–2), Moderate (TKC = 2/2–3) and Severe (TKC = 3/3–4). Eyes classified as TKC = 4 were excluded to prevent the potential deviation from extensive corneal scarring on the calculation of epithelial aberrations. The inclusion criteria for the FFKC group were: (1) KC diagnosis in the contralateral eye, (2) no corneal abnormalities on slit-lamp examination, (3) normal corneal topography, i.e. maximum keratometry (Kmax) < 47.2 diopters (D), inferior-superior difference (I-S value) at 6 mm ≤ 1.4 D, keratoconus percentage index (KISA% index) < 60% [18]. The identification criteria for normal corneas were as follows: (1) normal slit-lamp examination, (2) normal topography in both eyes, (3) BCVA ≥ 20/20, (4) being a candidate for laser vision correction (LVC) procedure as determined by a corneal specialist.

The exclusion criteria were: (1) symptoms or signs of dry eye, (2) wearing soft contact lenses within 2 weeks or rigid contact lenses within 4 weeks, (3) previous ocular surgery (including corneal crosslinking) or trauma, (4) other ocular condition or diseases, (5) poor cooperation during examination.

### Measurement procedure

All measurements were performed in a darkened room by one qualified operator using the MS-39 in accordance with the manufacturer's instructions. To minimize the potential impact of the diurnal variations of corneal curvature and thickness, all measurements were taken between 10:00 a.m. and 4:00 p.m. [19]. No eye drops were applied before the scan. First, with the chin on the chinrest and forehead against the forehead rest, the participants were asked to focus on the internal fixation light. Then, they were instructed to have a complete blink just before each measurement to ensure a smooth tear film over the cornea and keep their eyes open for a sufficient analysis area. The MS-39 marked green color to indicate a good acquisition for the keratoscopy quality, well-centeredness and coverage of the Placido-disk and the OCT topographies. Only valid readings were recorded, and each eye required three consecutive results to obtain an average for analysis.

### Instruments and parameters

The MS-39 (software version 4.0.1.8) is an AS-OCT combining spectral-domain optical coherence tomography (SD-OCT) with a 22-ring Placido-disk corneal topography. Each complete scan with the image field of 16 mm × 8 mm includes 25 meridional OCT images with 1024 A-scans per line and a Placido top-view image, providing high-resolution images of the anterior segment with the axial resolution of 3.6 μm in tissue and the transverse resolution of 35 μm in air.

**Fig. 1:**
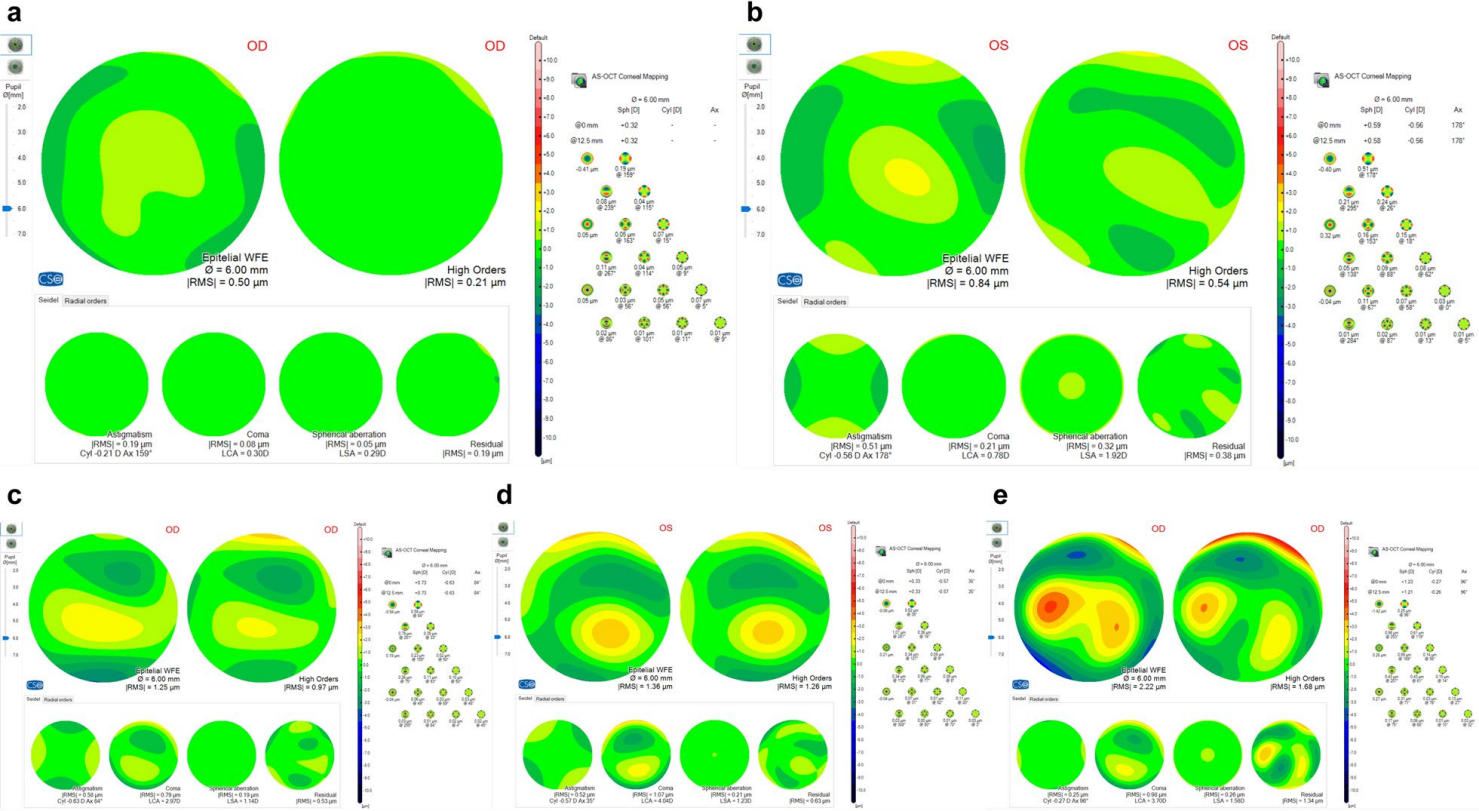
Sample images of epithelial wavefront analysis in MS-39. **a** Normal subject. **b** Patient with forme fruste keratoconus. **c** Patient with mild keratoconus. **d** Patient with moderate keratoconus. **e** Patient with severe keratoconus

The epithelial aberration analysis parameters were displayed by selecting ‘Epithelial wavefront analysis’ from the ‘Wavefront’ dropdown menu. It allowed us to analyze the wavefront generated by the measured epithelium using a set of 36 Zernike polynomials for the decomposition of total aberration. The 6-mm diameter range centered on the pupil was chosen for analysis. Sample images of each group are shown in Fig. 1. The root mean square (RMS) of total epithelial aberrations (higher- and lower-order aberrations), all HOAs (from the 3rd to the 7th Zernike polynomials), coma, trefoil, spherical aberration (SA), and secondary astigmatism (Ast II) were recorded.

In addition to epithelial aberrations, total corneal aberrations, epithelial thickness, and stromal thickness were measured and analyzed to provide a comprehensive comparison across epithelial maps, stromal maps, and wavefront profiles.

### Statistical analysis

Statistical analysis was performed with SPSS 25 (IBM Corporation, New York, USA), GraphPad Prism 10 (GraphPad software, California, USA) and Microsoft Office Excel 365 (Microsoft, Washington, USA). All continuous variables are presented as mean ± standard deviation (SD). Data normality was tested using the Shapiro–Wilk test (*P* > 0.05). To determine the differences among normal, FFKC, and KC groups, the normal distributed variables were compared using one-way analysis of variance (ANOVA) with Tukey’s or Games-Howell post-hoc tests, or else, the Kruskal–Wallis test with post-hoc multiple comparison was used. Differences were considered statistically significant when *P* < 0.05.

Receiver operating characteristic (ROC) curve and area under the ROC curve (AUC) were assessed to determine the discrimination ability of the indices in differentiating FFKC and KC from normal eyes. The optimal cut-off values for each of the indices with best sensitivity and specificity were calculated by Youden index [20]. Stepwise logistic regression was performed to develop the epithelial aberrations index (EAI), obtaining the most effective discriminant functions with maximal AUC value to diagnose FFKC (EAI-FFKC) and KC (EAI-KC). The discrimination performance of each parameter was graded according to the AUC value as follows: excellent (0.90–1.00), good (0.80–0.89), fair (0.70–0.79), poor (0.60–0.69) and fail (0.50–0.59) [21].

## Results

Ninety-one right eyes of 91 healthy participants and 235 eyes [87 FFKC and 148 KC (42 mild, 57 moderate, 49 severe)] of 235 KC patients were included and analyzed. Among the KC patients, 103 had bilateral KC and 45 had unilateral KC. Demographic information and corneal parameters of each group are shown in Table 1. When comparing the normal and FFKC groups, the FFKC group saw a younger age along with higher preponderance in males, which featured slightly lower central corneal thickness (CCT) (*P* < 0.001) thinnest corneal thickness (TCT) (*P* < 0.001); the remaining clinical characteristics (including spherical equivalent, cylinder, BCVA, keratometry) did not show statistically significant differences. The comparison of the normal and KC groups revealed significant differences for all clinical and tomographic variables. The KC groups consisted of younger participants and had a greater proportion of males. Across all KC groups, spherical equivalent, cylinder, BCVA, and posterior keratometry were lower, while average keratometry readings were higher. The KC groups also displayed notably thinner CCT and TCT compared to the normal group.

**Table 1:** Characteristics of healthy control group and the FFKC and KC groups

Parameter	Healthy controls	FFKC	KC	Mild KC	Moderate KC	Severe KC
N (eyes)	91	87	148	42	57	49
Age (years)	28.40 ± 6.49	24.51 ± 6.58^*^	25.06 ± 6.21^*^	25.31 ± 5.59^*^	25.16 ± 6.34^*^	24.73 ± 6.65^*^
Sex (M/F)	46/45	45/32^*^	90/58^*^	19/23	39/18^*^	31/16^*^
SE (D)	− 4.64 ± 1.58	− 4.08 ± 2.38	− 7.22 ± 4.25^*^	− 5.89 ± 2.82^*^	− 6.57 ± 4.04^*^	− 9.10 ± 4.89^*^
Cylinder (D)	− 0.85 ± 0.55	− 0.92 ± 0.64	− 3.65 ± 2.53^*^	− 3.04 ± 1.65^*^	− 3.70 ± 2.68^*^	− 4.11 ± 2.88^*^
BCVA (decimal)	1.00	1.00	0.63 ± 0.24^*^	0.81 ± 0.18^*^	0.65 ± 0.19^*^	0.40 ± 0.18^*^
Average K (D)	43.34 ± 1.15	43.16 ± 1.16	48.73 ± 5.77^*^	45.06 ± 1.75^*^	46.64 ± 2.59^*^	54.32 ± 6.54^*^
Posterior K (D)	− 6.22 ± 0.17	− 6.28 ± 0.26	− 7.40 ± 1.07^*^	− 6.70 ± 0.31^*^	− 7.08 ± 0.53^*^	− 8.38 ± 1.25^*^
CCT (μm)	538.76 ± 32.09	514.90 ± 26.89^*^	461.80 ± 43.52^*^	487.29 ± 35.89^*^	469.23 ± 32.24^*^	430.67 ± 42.94^*^
TCT (μm)	533.07 ± 32.43	508.24 ± 27.26^*^	453.45 ± 44.49^*^	478.28 ± 38.97^*^	463.13 ± 30.68^*^	420.54 ± 43.19^*^

*FFKC* = forme fruste keratoconus group; *KC* = keratoconus group; *SE* = spherical equivalent; *D* = diopter; *cylinder* = cylindrical power; *BCVA* = best-corrected visual acuity; *average K* = average keratometry; *posterior K* = posterior keratometry; *CCT* = central corneal thickness; *TCT* = thinnest corneal thickness

Values are presented as mean ± standard deviation. Statistical comparisons were performed using one-way ANOVA with Games-Howell post-hoc test. FFKC, KC, mild KC, moderate KC, severe KC groups were compared to the healthy control group

^*^Statistically significant (*P* < 0.05) compared to the normal group

**Fig. 2:**
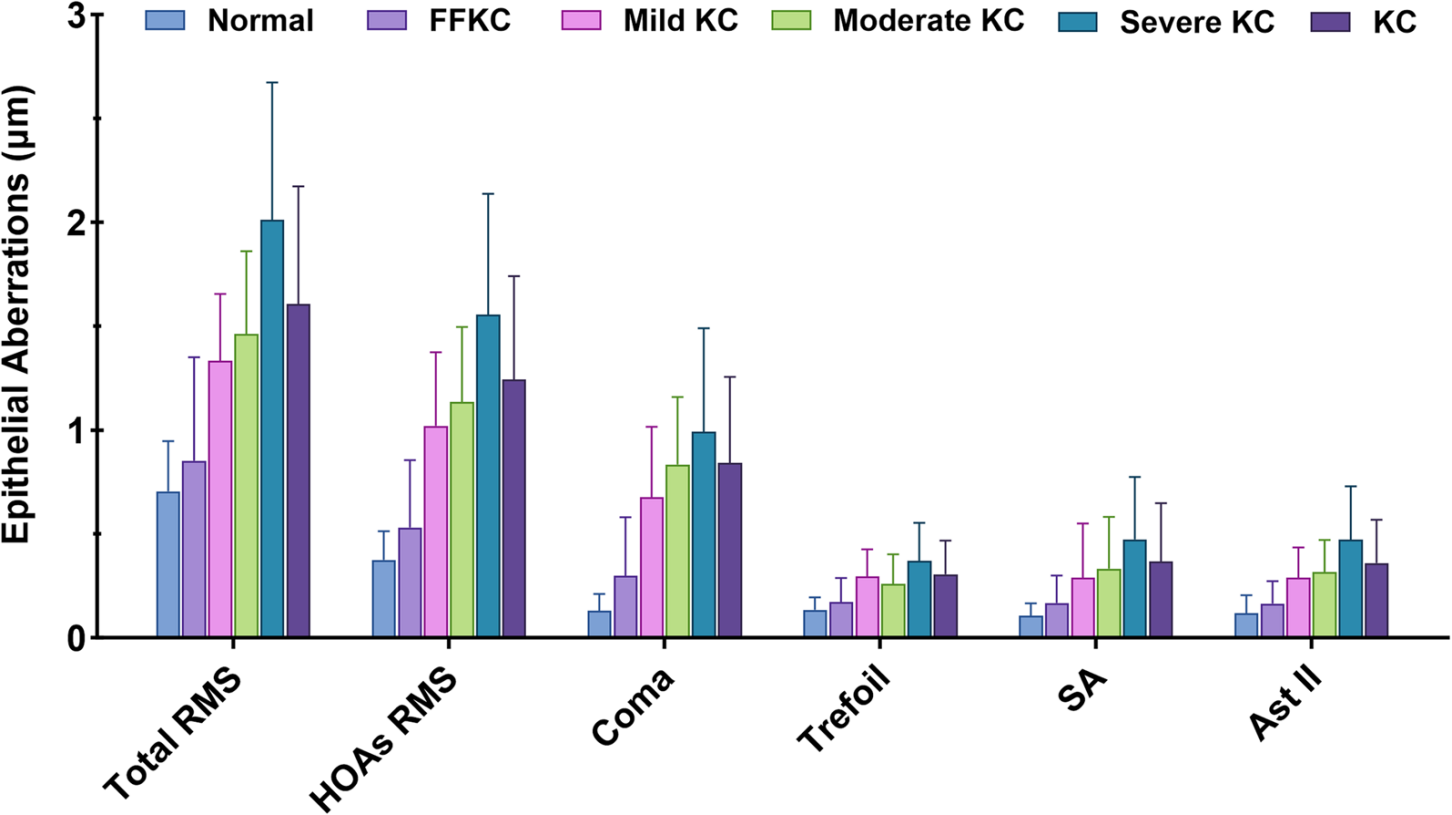
Histogram showing mean values and standard deviations of epithelial aberrations diagnostic indices in normal, forme fruste keratoconus (FFKC) and keratoconus (KC) groups. RMS, root mean square; HOAs, high-order aberrations; SA, spherical aberration; Ast II, secondary astigmatism

Table 2 and Fig. 2 demonstrate the mean values of epithelial aberrations for a 6-mm pupil in each group. All epithelial wavefront parameters were significantly different among the six groups (*P* < 0.001). When comparing the normal and FFKC groups, all epithelial wavefront values were significantly higher in FFKC (*P* < 0.011). Also, all epithelial wavefront parameters were significantly higher (*P* < 0.001) in the KC group when compared to the normal group. The same trend was observed for all stages of KC.

**Table 2:** Epithelial aberrations of normal, FFKC and KC groups

Parameters	Normal (n = 91)	FFKC (n = 87)	Mild KC (n = 42)	Moderate KC (n = 57)	Severe KC (n = 49)	KC (n = 148)
Total RMS	0.705 ± 0.242	0.853 ± 0.497^*^	1.331 ± 0.324^*^	1.456 ± 0.403^*^	2.013 ± 0.661^*^	1.605 ± 0.567^*^
HOAs RMS	0.374 ± 0.138	0.531 ± 0.325^*^	1.021 ± 0.353^*^	1.130 ± 0.360^*^	1.554 ± 0.583^*^	1.239 ± 0.499^*^
Coma	0.131 ± 0.080	0.298 ± 0.282^*^	0.677 ± 0.339^*^	0.835 ± 0.321^*^	0.992 ± 0.498^*^	0.842 ± 0.412^*^
Trefoil	0.135 ± 0.060	0.172 ± 0.117^*^	0.296 ± 0.130^*^	0.260 ± 0.139^*^	0.370 ± 0.184^*^	0.307 ± 0.160^*^
Spherical aberration	0.106 ± 0.060	0.167 ± 0.133^*^	0.220 ± 0.321^*^	0.283 ± 0.296^*^	0.415 ± 0.355^*^	0.309 ± 0.333^*^
Secondary astigmatism	0.118 ± 0.088	0.164 ± 0.108^*^	0.290 ± 0.145^*^	0.311 ± 0.151^*^	0.472 ± 0.257^*^	0.358 ± 0.207^*^

*Normal* = normal group; *FFKC* = forme fruste keratoconus group; *KC =* keratoconus group; *RMS* = root mean square; *HOAs* = higher-order aberrations

Values are presented as mean ± standard deviation

^*^Statistically significant (*P* < 0.05) compared to the normal group

**Fig. 3:**
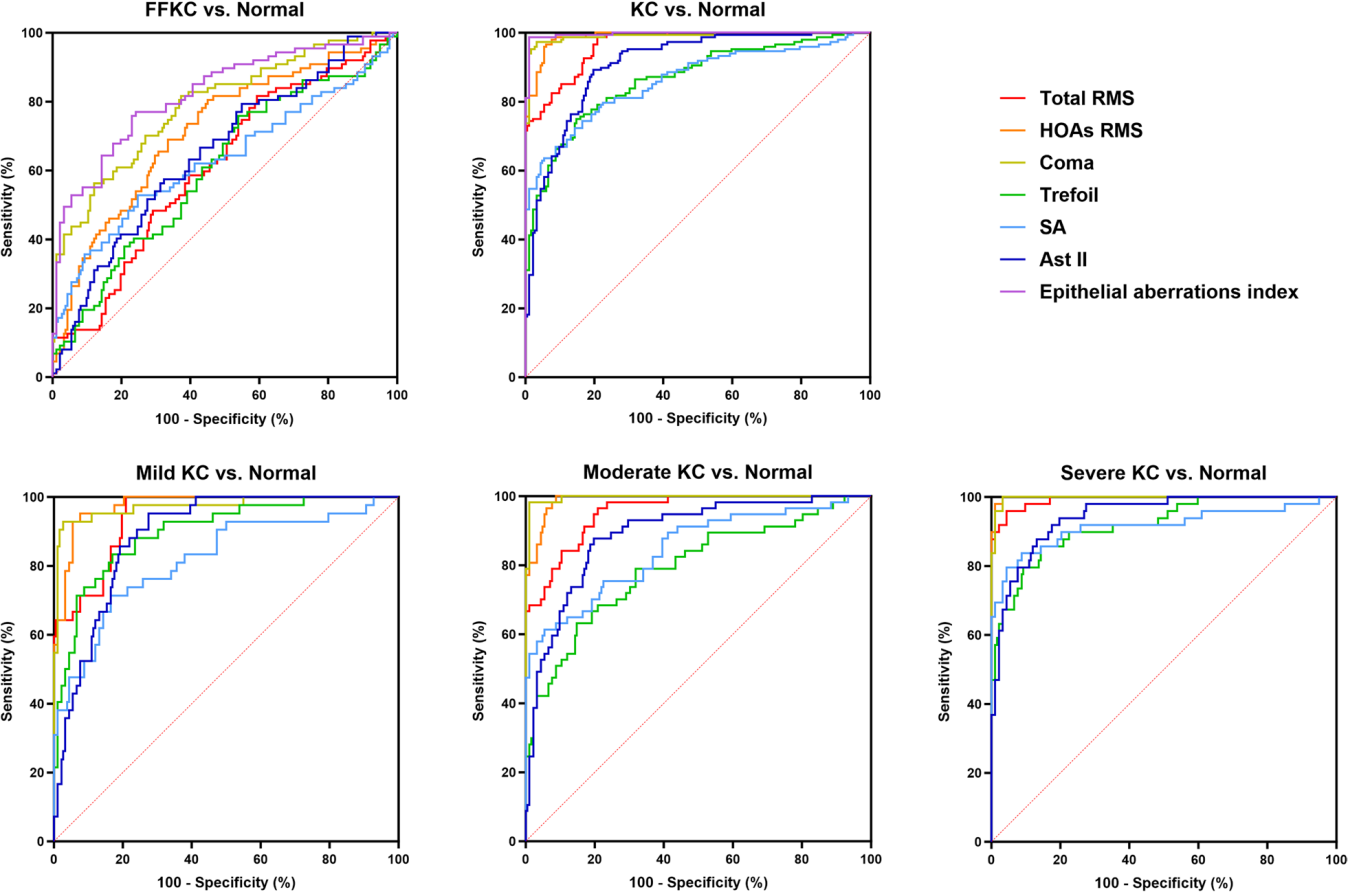
Receiver operating characteristic (ROC) curve of the epithelial aberrations diagnostic indices for forme fruste keratoconus (FFKC), keratoconus (KC), mild KC, moderate KC, severe KC groups vs. normal group. Epithelial aberrations index in FFKC vs. Normal = − 4.761 × HOAs RMS + 14.467 × Coma + 7.972 × SA − 1.697. Epithelial aberrations index in KC vs. Normal = 4.355 × HOAs RMS + 16.55 × Coma + 10.293 × SA − 9.389. RMS, root mean square; HOAs, high-order aberrations; SA, spherical aberration; Ast II, secondary astigmatism

Figure 3 illustrates the ROC curves used to distinguish FFKC and KC from normal eyes. Table 3 displays the AUC values and optimal cut-off values that yield the highest sensitivity and specificity for the diagnostic indices of epithelial aberrations. For the diagnosis of FFKC, all diagnostic indices were statistically significant (*P* < 0.013). The EAI for FFKC (EAI-FFKC = − 4.761 × HOAs RMS + 14.467 × Coma + 7.972 × SA − 1.697) exhibited the highest discrimination power by attaining AUC value of 0.822 with 77.0% sensitivity and 75.8% specificity. Epithelial HOAs RMS and coma showed AUC values of 0.714 (80.5% sensitivity and 54.9% specificity) and 0.788 (81.6% sensitivity and 62.6% specificity), respectively. For the diagnosis of KC, all diagnostic indices were statistically significant (*P* < 0.001). The EAI for KC (EAI-KC = 4.355 × HOAs RMS + 16.55 × Coma + 10.293 × SA − 9.389) exhibited the highest discriminatory power by attaining AUC value of 0.996 with 98.6% sensitivity and 98.9% specificity. The epithelial HOAs RMS and coma presented excellent discrimination performance with AUC values of 0.989 (95.9% sensitivity and 94.5% specificity) and 0.990 (97.3% sensitivity and 96.7% specificity), respectively. Across various stages of KC groups, the AUC values of the epithelial HOAs RMS (0.976–0.998) and coma (0.974–0.997) were consistently the highest, followed by RMS of higher and lower-order aberrations (total RMS, 0.944–0.992), Ast II (0.890–0.944), SA (0.819–0.915), whereas the lowest were obtained by trefoil (0.793–0.918).

**Table 3:** Diagnostic indices of epithelial aberrations to differentiate FFKC from normal eyes and KC from normal eyes

Parameters	*P*	AUC	Cut-off (μm)	Sensitivity (%)	Specificity (%)	Cohen’s d
FFKC						
Total RMS	0.008	0.612	0.577	81.6	40.7	0.390
HOAs RMS	< 0.001	0.714	0.335	80.5	54.9	0.249
Coma	< 0.001	0.788	0.125	81.6	62.6	0.207
Trefoil	0.013	0.605	0.115	75.9	46.2	0.093
Spherical aberration	0.002	0.630	0.135	52.9	74.7	0.103
Secondary astigmatism	< 0.001	0.651	0.115	57.5	67.0	0.099
EAI-FFKC	< 0.001	0.822	-0.417	77.0	75.8	2.367
KC						
Total RMS	< 0.001	0.964	0.857	98.6	80.0	0.471
HOAs RMS	< 0.001	0.989	0.640	95.9	94.5	0.403
Coma	< 0.001	0.990	0.258	97.3	96.7	0.330
Trefoil	< 0.001	0.865	0.188	75.0	84.6	0.133
Spherical aberration	< 0.001	0.861	0.192	66.9	91.2	0.225
Secondary astigmatism	< 0.001	0.908	0.152	89.2	80.2	0.173
EAI-KC	< 0.001	0.996	-0.109	98.6	98.9	4.015
Mild KC						
Total RMS	< 0.001	0.944	0.857	100.0	80.0	0.273
HOAs RMS	< 0.001	0.976	0.583	95.2	92.3	0.231
Coma	< 0.001	0.974	0.275	92.9	96.7	0.203
Trefoil	< 0.001	0.899	0.182	83.3	82.4	0.089
Spherical aberration	< 0.001	0.819	0.162	71.4	81.3	0.156
Secondary astigmatism	< 0.001	0.890	0.128	95.2	72.5	0.110
Moderate KC						
Total RMS	< 0.001	0.954	0.868	96.5	80.0	0.314
HOAs RMS	< 0.001	0.990	0.552	100.0	91.2	0.251
Coma	< 0.001	0.996	0.327	98.2	98.9	0.211
Trefoil	< 0.001	0.793	0.188	63.2	84.6	0.101
Spherical aberration	< 0.001	0.846	0.210	61.4	94.5	0.163
Secondary astigmatism	< 0.001	0.891	0.153	87.7	80.2	0.119
Severe KC						
Total RMS	< 0.001	0.992	1.225	95.9	95.6	0.440
HOAs RMS	< 0.001	0.998	0.818	100.0	96.7	0.365
Coma	< 0.001	0.997	0.258	100.0	96.7	0.304
Trefoil	< 0.001	0.918	0.195	85.7	85.7	0.120
Spherical aberration	< 0.001	0.915	0.227	79.6	95.6	0.186
Secondary astigmatism	< 0.001	0.944	0.175	91.8	82.4	0.169

*AUC* = area under the receiver operating characteristic curve; *FFKC* = forme fruste keratoconus group; *KC* = keratoconus group; *RMS* = root mean square; *HOAs* = higher-order aberrations; *EAI* = epithelial aberration index

Epithelial aberration index was developed by stepwise logistic regression

To further understand early disease progression, we compared epithelial aberration parameters between FFKC and mild KC groups, as shown in Table 4. All parameters were statistically significant (*P* < 0.001). HOAs RMS demonstrated the highest discriminative ability (AUC = 0.906) with 92.9% sensitivity and 75.9% specificity. Total RMS also showed excellent performance (AUC = 0.880) with 100.0% sensitivity and 66.7% specificity. Coma showed good diagnostic ability with AUC values of 0.856. The diagnostic performance of total corneal aberrations, epithelial thickness, and stromal thickness were presented in Supplemental Tables 1–3.

**Table 4:** Diagnostic accuracy of epithelial aberrations in differentiating FFKC from mild KC

Parameters	*P*	AUC	Cut-off (μm)	Sensitivity (%)	Specificity (%)
Total RMS	< 0.001	0.880	0.853	100.0	66.7
HOAs RMS	< 0.001	0.906	0.655	92.9	75.9
Coma	< 0.001	0.856	0.430	78.6	81.6
Trefoil	< 0.001	0.828	0.243	69.1	88.5
Spherical aberration	< 0.001	0.681	0.162	71.4	58.6
Secondary astigmatism	< 0.001	0.782	0.142	90.5	58.6

*AUC* = area under the receiver operating characteristic curve; *FFKC* = forme fruste keratoconus group; *KC* = keratoconus group; *RMS* = root mean square; *HOAs* = higher-order aberrations

## Discussion

AS-OCT provides a non-invasive, non-contact imaging method in the field of ophthalmology. Its rapid scan speed minimizes the effects of eye-movements and has an advantage over some Scheimpflug-based equipment. Reinstein et al. [6, 7, 15] have described the epithelial compensatory mechanism due to stromal irregularities in KC, providing a perspective on the detection of this corneal ectatic disease. Several recent studies have utilized the OCT technology to distinguish KC from healthy corneas by analyzing the corneal epithelium thickness profile [5, 8, 10, 22–26]. The results of this study showed that FFKC and KC had higher epithelial aberrations compared to normal eyes, several parameters exhibited the ability for diagnosing FFKC and KC.

To identify suitable candidates for LVC surgery, it is crucial to screen for early-stage KC during preoperative assessments, given that it is a major risk factor for post-LVC ectasia [27, 28]. Previous studies had attempted to diagnose FFKC using various metrics such as corneal thickness, topographical, biomechanical, or aberrations data, yet this remains challenging for ophthalmologists. Epithelial thickness maps may help identify FFKC in patients who have an otherwise normal topography. Temstet et al. reported that epithelial thickness in the thinnest corneal zone had 0.79 of AUC in discrimination of FFKC [29]. However, in Toprak et al.’s study, this parameter showed no statistically significant ability for distinguishing FFKC. Thinnest pachymetry and stromal thicknesses [center, nasal, superior, inferior, inferior-nasal (IN), superior-nasal (SN), inferior-temporal (IT), superior-temporal (ST), and at the thinnest epithelial point] in discrimination of eyes with FFKC from normal controls (AUC ranges between 0.614 and 0.712) [10]. Similar results were also obtained in our study. Supplemental Table 2 presents the diagnostic performance of epithelial thickness across different corneal regions, with AUC values for FFKC ranging from 0.532 to 0.717. Supplemental Table 3 presents the diagnostic performance of stromal thickness measurements, with AUC values ranging from 0.660 to 0.755 for FFKC. Koh et al. [30] conducted a study measuring ocular and corneal HOAs in pre-topographic KC (equivalent to 'FFKC' in our study) and evaluated the ability of wavefront data to distinguish FFKC from normal eyes. Their results showed all ocular and corneal wavefront parameters were not different between FFKC and control groups, whereas our study showed that all epithelial wavefront parameters (total RMS, HOAs RMS, coma, trefoil, SA, Ast II) were significantly higher in the FFKC group. This discrepancy suggests that while the ocular and total cornea remain optically unchanged, the epithelial wavefront analysis could detect variations in the pathological cornea from the earliest stage of KC progression. In the diagnosis of FFKC, Koh’s study [30] indicated that ocular wavefront data were insufficient for detecting FFKC, but the corneal HOAs RMS (AUC: 0.781, sensitivity: 100.0%, specificity: 47.0%) and corneal coma (AUC: 0.735, sensitivity: 73.0%, specificity: 73.0%) exhibited a potential to discriminate between FFKC and control eyes. Saad et al. [31] indicated that corneal coma had an AUC of 0.778 with 71.0% sensitivity and 80.0% specificity. According to our data, the epithelial HOAs RMS (AUC: 0.714, sensitivity: 80.5%, specificity: 54.9%) and epithelial coma (AUC: 0.788, sensitivity: 81.6%, specificity: 62.6%) demonstrated high discriminatory ability and also had the potential to differentiate FFKC from healthy corneas. To optimize diagnostic accuracy, we employed stepwise logistic regression to construct the EAI (EAI-FFKC), a discriminant function for distinguishing FFKC from healthy corneas. The EAI-FFKC showed the best discriminatory power by AUC value of 0.822 with 77.0% sensitivity and 75.8% specificity. This multi-parameter diagnostic approach, combining epithelial HOAs RMS, coma, and SA, demonstrated greater diagnostic accuracy than any single parameter in distinguishing FFKC from normal corneas, improving both sensitivity and specificity. Previous studies investigated corneal wavefront indices generated from Scheimpflug, Placido, and Hartmann–Shack based devices with acceptable validity for differentiating normal corneas from early subclinical keratoconus (SKCN). The front Baiocchi-Calossi-Versaci (BCV) index from Sirius was the most accurate parameter for diagnosing SKCN (AUC = 0.887), followed by vertical coma (AUC = 0.857) with Pentacam and OPD-Scan III (AUC = 0.857) [32]. An eye was diagnosed with SKCN if it had at least three of the following abnormal topographic criteria: a skewed asymmetric bow tie, central or inferior steepening in anterior/posterior elevation height. SKCN is the later form of FFKC, so there is a slightly higher AUC value [33, 34].

Supplemental Table 1 shows the diagnostic performance of total corneal aberrations. In the FFKC group, the AUC values for epithelial HOAs RMS and coma were consistently higher than those of total corneal aberrations (epithelial vs. total: HOAs RMS, 0.714 vs. 0.701; coma, 0.788 vs. 0.728). In contrast, epithelial trefoil and Ast II yielded lower AUCs compared to total corneal aberrations (epithelial vs. total: trefoil, 0.605 vs. 0.680; Ast II, 0.651 vs. 0.680). A similar trend was observed in the mild KC group (HOAs RMS, 0.974 vs. 0.957; coma, 0.976 vs. 0.958; trefoil, 0.899 vs. 0.951; Ast II, 0.890 vs. 0.952). Additionally, our comparison between FFKC and mild KC groups provided further insight into early KC progression. The AUC values of all parameters were higher when distinguishing between FFKC and mild KC than between FFKC and normal corneas, suggesting that FFKC eyes more closely resemble healthy eyes than KC eyes.

Mohammadpour et al. found that the natural shape of the cornea changes over time, causing visual distortions and increasing aberrations [35]. Salman et al. believe that analyzing the different characteristics of aberrations can improve diagnostic accuracy, especially in the early stages of KC [36]. Previous studies [30, 36–39] reported that higher levels of ocular and corneal aberrations were exhibited in clinical KC and topographic KC compared to normal eyes. Our data also showed that all epithelial wavefront parameters of KC were significantly higher than normal eyes. Interestingly, as KC progressed through stages from FFKC to mild, moderate, and severe, there were corresponding increases in all types of epithelial aberrations. This revealed that the epithelial compensation mechanism was presented throughout the entire disease progression of KC. Concerning KC diagnosis, Saad et al. [31] reported that corneal coma and trefoil reached AUC values of 0.988 (sensitivity: 98.0%, specificity: 99.0%) and 0.960 (sensitivity: 94.0%, specificity: 94.0%), respectively. Kandel et al. found that corneal aberration increases with KC severity, and indicators like corneal asymmetry index (SIb) and coma effectively detect subclinical KC (AUC > 0.90) [40]. Although the change of corneal thickness contributes to diagnosis (AUC = 0.77–0.94), its sensitivity and specificity are lower than those of aberration parameters (AUC = 0.71–0.95) [41]. Vertical coma consistently exhibited the greatest magnitude of HOA across all irregular cornea types [42]. In this study, the epithelial HOAs RMS and coma demonstrated excellent diagnostic accuracy, achieving AUC values exceeding 0.989, with very high sensitivity (95.9%–97.3%) and specificity (94.5%–96.7%). These parameters also effectively diagnosed the mild stage of KC, maintaining high AUC values of 0.976 and 0.974, with high sensitivity of 92.9% and 95.2%, specificity of 92.3% and 96.7%, respectively. For KC, most stromal parameters showed AUC values between 0.70 and 0.90, which were generally lower than those observed for epithelial aberrations. Given that KC may manifest in different corneal regions among individuals, epithelial or stromal thickness alone may be insufficient for reliable diagnosis. In contrast, epithelial wavefront analysis provides a comprehensive assessment of the corneal epithelium and offers broader applicability across various KC presentations. As a result, the epithelial HOAs RMS and coma could be independent indicators for discriminating KC eyes from healthy corneas. Elkitkat et al. demonstrated that BCV indices differed significantly between healthy individuals and those with KC using MS-39 (*P* < 0.001) [43]. The analysis yielded an AUC value of 0.994, along with a sensitivity of 97.7% and a specificity of 98.4%. Moreover, the EAI-KC discriminant function constructed by the epithelial HOAs RMS, coma and SA, attained the highest AUC value of 0.996 with 98.6% sensitivity and 98.9% specificity for diagnosing KC. Only the EAI-KC (AUC = 0.996) exceeds BCV of 0.994, indicating exceptional KC diagnostic performance. Coma (AUC = 0.990) and HOAs RMS (AUC = 0.989) are slightly lower, while total RMS, trefoil, SA, and Ast II fall below the value. The preference for "Epithelial Wavefront Analysis" arises from its ability to provide a comprehensive and sensitive assessment of the corneal epithelium, which is particularly valuable in the early detection of KC. The epithelium undergoes compensatory remodeling—thinning over areas of stromal protrusion and thickening in surrounding regions. This adaptive behavior allows epithelial aberrations to highlight subtle, region-specific abnormalities that may be missed by stromal thickness maps, especially given the variable presentation of KC across different individuals. Furthermore, epithelial wavefront analysis offers a user-friendly diagnostic approach, enhancing its clinical utility for detecting early or subclinical cases.

Our study is not without limitations. The MS-39 device has been shown to produce reproducible measurements in both healthy and keratoconic eyes [44–47]. However, since the repeatability of epithelial aberrations has not yet been investigated, we used mean values from three consecutive measurements to obtain reliable data [48]. Moreover, the EAI discriminant function was not cross-validated with an independent dataset due to the small sample size, limiting its generalizability. Future studies should also investigate the longitudinal changes of these indices and their potential to predict disease progression and exploring the combination of epithelial aberration analysis with other technologies.

## Conclusion

This is the first study to provide data on the use of epithelial aberrations for KC diagnosis. Epithelial wavefront analysis could effectively detect the abnormal corneal epithelial changes from the very early to severe stages of KC. The analysis of epithelial aberrations could serve as novel diagnostic parameters for the detection of KC and FFKC.

## Supplementary Information

Supplementary material 1.

## Data Availability

All data analyzed during this study are included in this published article.

## Abbreviations

KC: Keratoconus
AS-OCT: Anterior segment optical coherence tomography
FFKC: Forme fruste keratoconus
BCVA: Best-corrected visual acuity
TKC: Topographical keratoconus classification
Kmax: Maximum keratometry
I-S value: Inferior-superior difference value
KISA% index: Keratoconus percentage index
SD-OCT: Spectral-domain optical coherence tomography
ROC: Receiver operating characteristic
AUC: Area under the receiver operating characteristic curve
EAI: Epithelial aberrations index
Total RMS: Root mean square of higher and lower-order aberrations
HOAs RMS: Root mean square of higher-order aberrations
SA: Spherical aberration
Ast II: Secondary astigmatism
D: Diopter
CCT: Central corneal thickness
TCT: Thinnest corneal thickness
LVC: Laser vision correction

## References

[1] Rabinowitz YS. Keratoconus. Surv Ophthalmol. 1998;42(4):297–319. 9493273 10.1016/s0039-6257(97)00119-7

[2] Sahebjada S, Fenwick EK, Xie J, Snibson GR, Daniell MD, Baird PN. Impact of keratoconus in the better eye and the worse eye on vision-related quality of life. Invest Ophthalmol Vis Sci. 2014;55(1):412–6. 24398095 10.1167/iovs.13-12929

[3] Santodomingo-Rubido J, Carracedo G, Suzaki A, Villa-Collar C, Vincent SJ, Wolffsohn JS. Keratoconus: an updated review. Cont Lens Anterior Eye. 2022;45(3):101559. 34991971 10.1016/j.clae.2021.101559

[4] Gomes JAP, Tan D, Rapuano CJ, Belin MW, Ambrósio R, Guell JL, et al. Global consensus on keratoconus and ectatic diseases. Cornea. 2015;34(4):359–69. 25738235 10.1097/ICO.0000000000000408

[5] Li Y, Tan O, Brass R, Weiss JL, Huang D. Corneal epithelial thickness mapping by fourier-domain optical coherence tomography in normal and keratoconic eyes. Ophthalmology. 2012;119(12):2425–33. 22917888 10.1016/j.ophtha.2012.06.023 PMC3514625

[6] Reinstein DZ, Archer TJ, Gobbe M, Silverman RH, Coleman DJ. Epithelial thickness in the normal cornea: three-dimensional display with very high frequency ultrasound. J Refract Surg. 2008;24(6):571–81. 18581782 10.3928/1081597X-20080601-05 PMC2592549

[7] Reinstein DZ, Gobbe M, Archer TJ, Silverman RH, Coleman DJ. Epithelial, stromal, and total corneal thickness in keratoconus: three-dimensional display with artemis very-high frequency digital ultrasound. J Refract Surg. 2010;26(4):259–71. 20415322 10.3928/1081597X-20100218-01 PMC3655809

[8] Haque S, Simpson T, Jones L. Corneal and epithelial thickness in keratoconus: a comparison of ultrasonic pachymetry, Orbscan II, and optical coherence tomography. J Refract Surg. 2006;22(5):486–93. 16722488 10.3928/1081-597X-20060501-11

[9] Li Y, Xu Z, Liu Q, Wang Y, Lin K, Xia J, et al. Relationship between corneal biomechanical parameters and corneal sublayer thickness measured by Corvis ST and UHR-OCT in keratoconus and normal eyes. Eye Vis (Lond). 2021;8:2. 33419485 10.1186/s40662-020-00225-z PMC7796648

[10] Toprak I, Vega A, Alió Del Barrio JL, Espla E, Cavas F, Alió JL. Diagnostic value of corneal epithelial and stromal thickness distribution profiles in forme fruste keratoconus and subclinical keratoconus. Cornea. 2021;40(1):61–72. 32769675 10.1097/ICO.0000000000002435

[11] Gordon-Shaag A, Millodot M, Ifrah R, Shneor E. Aberrations and topography in normal, keratoconus-suspect, and keratoconic eyes. Optom Vis Sci. 2012;89(4):411–8. 22311193 10.1097/OPX.0b013e318249d727

[12] Naderan M, Jahanrad A, Farjadnia M. Ocular, corneal, and internal aberrations in eyes with keratoconus, forme fruste keratoconus, and healthy eyes. Int Ophthalmol. 2018;38(4):1565–73. 28647782 10.1007/s10792-017-0620-5

[13] Maeda N, Fujikado T, Kuroda T, Mihashi T, Hirohara Y, Nishida K, et al. Wavefront aberrations measured with Hartmann-Shack sensor in patients with keratoconus. Ophthalmology. 2002;109(11):1996–2003. 12414405 10.1016/s0161-6420(02)01279-4

[14] Franco J, White CA, Kruh JN. Analysis of compensatory corneal epithelial thickness changes in keratoconus using corneal tomography. Cornea. 2020;39(3):298–302. 31567631 10.1097/ICO.0000000000002156

[15] Reinstein DZ, Archer TJ, Gobbe M. Corneal epithelial thickness profile in the diagnosis of keratoconus. J Refract Surg. 2009;25(7):604–10. 19662917 10.3928/1081597X-20090610-06

[16] Rocha KM, Perez-Straziota CE, Stulting RD, Randleman JB. SD-OCT analysis of regional epithelial thickness profiles in keratoconus, postoperative corneal ectasia, and normal eyes. J Refract Surg. 2013;29(3):173–9. 23446013 10.3928/1081597X-20130129-08 PMC4123636

[17] Mas Tur V, MacGregor C, Jayaswal R, O’Brart D, Maycock N. A review of keratoconus: diagnosis, pathophysiology, and genetics. Surv Ophthalmol. 2017;62(6):770–83. 28688894 10.1016/j.survophthal.2017.06.009

[18] Henriquez MA, Hadid M, Izquierdo L Jr. A systematic review of subclinical keratoconus and forme fruste keratoconus. J Refract Surg. 2020;36(4):270–9. 32267959 10.3928/1081597X-20200212-03

[19] Read SA, Collins MJ. Diurnal variation of corneal shape and thickness. Optom Vis Sci. 2009;86(3):170–80. 19182699 10.1097/OPX.0b013e3181981b7e

[20] Ruopp MD, Perkins NJ, Whitcomb BW, Schisterman EF. Youden index and optimal cut-point estimated from observations affected by a lower limit of detection. Biom J. 2008;50(3):419–30. 18435502 10.1002/bimj.200710415 PMC2515362

[21] Nahm FS. Receiver operating characteristic curve: overview and practical use for clinicians. Korean J Anesthesiol. 2022;75(1):25–36. 35124947 10.4097/kja.21209 PMC8831439

[22] Pircher N, Schwarzhans F, Holzer S, Lammer J, Schmidl D, Bata AM, et al. Distinguishing keratoconic eyes and healthy eyes using ultrahigh-resolution optical coherence tomography-based corneal epithelium thickness mapping. Am J Ophthalmol. 2018;189:47–54. 29458037 10.1016/j.ajo.2018.02.006

[23] Salomão MQ, Hofling-Lima AL, Gomes Esporcatte LP, Correa FF, Meneses EF, Li Y, et al. Corneal ectasia detection by epithelial pattern standard deviation from OCT. J Cataract Refract Surg. 2023;49(2):190–4. 36201664 10.1097/j.jcrs.0000000000001066

[24] Hu L, Li Y, Liu Q, Xu Z, Gu J, Li A, et al. Corneal vertical and horizontal thickness profiles generated by UHR-OCT for suspected and subclinical keratoconus diagnosis. J Refract Surg. 2021;37(7):438–45. 34236909 10.3928/1081597X-20210330-01

[25] Kanellopoulos AJ, Asimellis G. OCT corneal epithelial topographic asymmetry as a sensitive diagnostic tool for early and advancing keratoconus. Clin Ophthalmol. 2014;8:2277–87. 25429197 10.2147/OPTH.S67902 PMC4242699

[26] Catalan S, Cadarso L, Esteves F, Salgado-Borges J, Lopez M, Cadarso C. Assessment of corneal epithelial thickness in asymmetric keratoconic eyes and normal eyes using Fourier domain optical coherence tomography. J Ophthalmol. 2016;2016:5697343. 27379181 10.1155/2016/5697343 PMC4917694

[27] Randleman JB, Russell B, Ward MA, Thompson KP, Stulting RD. Risk factors and prognosis for corneal ectasia after LASIK. Ophthalmology. 2003;110(2):267–75. 12578766 10.1016/S0161-6420(02)01727-X

[28] Binder PS. Analysis of ectasia after laser in situ keratomileusis: risk factors. J Cataract Refract Surg. 2007;33(9):1530–8. 17720066 10.1016/j.jcrs.2007.04.043

[29] Temstet C, Sandali O, Bouheraoua N, Hamiche T, Galan A, El Sanharawi M, et al. Corneal epithelial thickness mapping using Fourier-domain optical coherence tomography for detection of form fruste keratoconus. J Cataract Refract Surg. 2015;41(4):812–20. 25840306 10.1016/j.jcrs.2014.06.043

[30] Koh S, Inoue R, Maeno S, Mihashi T, Maeda N, Jhanji V, et al. Characteristics of higher-order aberrations in different stages of keratoconus. Eye Contact Lens. 2022;48(6):256–60. 35333804 10.1097/ICL.0000000000000897

[31] Saad A, Gatinel D. Evaluation of total and corneal wavefront high order aberrations for the detection of forme fruste keratoconus. Invest Ophthalmol Vis Sci. 2012;53(6):2978–92. 22427590 10.1167/iovs.11-8803

[32] Heidari Z, Mohammadpour M, Hashemi H, Jafarzadehpur E, Moghaddasi A, Yaseri M, et al. Early diagnosis of subclinical keratoconus by wavefront parameters using Scheimpflug, Placido and Hartmann-Shack based devices. Int Ophthalmol. 2020;40(7):1659–71. 32219617 10.1007/s10792-020-01334-3

[33] Maeda N, Klyce SD, Smolek MK, Thompson HW. Automated keratoconus screening with corneal topography analysis. Invest Ophthalmol Vis Sci. 1994;35(6):2749–57. 8188468

[34] Rabinowitz YS, McDonnell PJ. Computer-assisted corneal topography in keratoconus. Refract Corneal Surg. 1989;5(6):400–8. 2488838

[35] Mohammadpour M, Heidari Z, Mohammad-Rabei H, Jafarzadehpur E, Jabbarvand M, Hashemi H, et al. Correlation of higher order aberrations and components of astigmatism in myopic refractive surgery candidates. J Curr Ophthalmol. 2016;28(3):112–6. 27579454 10.1016/j.joco.2016.04.007 PMC4992119

[36] Salman A, Kailani O, Ghabra M, Omran R, Darwish TR, Shaaban R, et al. Corneal higher order aberrations by Sirius topography and their relation to different refractive errors. BMC Ophthalmol. 2023;23(1):104. 36927406 10.1186/s12886-023-02841-4 PMC10018888

[37] Jafri B, Li X, Yang H, Rabinowitz YS. Higher order wavefront aberrations and topography in early and suspected keratoconus. J Refract Surg. 2007;23(8):774–81. 17985796 10.3928/1081-597X-20071001-06

[38] Alió JL, Shabayek MH. Corneal higher order aberrations: a method to grade keratoconus. J Refract Surg. 2006;22(6):539–45. 16805116 10.3928/1081-597X-20060601-05

[39] Lim L, Wei RH, Chan WK, Tan DT. Evaluation of higher order ocular aberrations in patients with keratoconus. J Refract Surg. 2007;23(8):825–8. 17985803 10.3928/1081-597X-20071001-13

[40] Kandel S, Chaudhary M, Mishra SK, Joshi ND, Subedi M, Puri PR, et al. Evaluation of corneal topography, pachymetry and higher order aberrations for detecting subclinical keratoconus. Ophthalmic Physiol Opt. 2022;42(3):594–608. 35147226 10.1111/opo.12956

[41] Heidari Z, Jafarzadehpour E, Mohammadpour M, Hashemi H. Best indices of dual Scheimpflug/Placido tomographer for keratoconus detection. Int Ophthalmol. 2023;43(4):1353–62. 36149621 10.1007/s10792-022-02533-w

[42] Martínez-Pérez C, Santodomingo-Rubido J, Villa-Collar C, Bodas-Romero J, Carracedo G, Serramito Blanco M, et al. Corneal higher-order aberrations in different types of irregular cornea. J Optom. 2024;17(4):100522. 39317099 10.1016/j.optom.2024.100522 PMC11462483

[43] Elkitkat RS, Rifay Y, Gharieb HM, Ziada HEA. Accuracy of the indices of MS-39 anterior segment optical coherence tomography in the diagnosis of keratoconic corneas. Eur J Ophthalmol. 2022;32(4):2116–24. 34841916 10.1177/11206721211063720

[44] Vega-Estrada A, Mimouni M, Espla E, Alió Del Barrio J, Alio JL. Corneal epithelial thickness intrasubject repeatability and its relation with visual limitation in keratoconus. Am J Ophthalmol. 2019;200:255–62. 30689987 10.1016/j.ajo.2019.01.015

[45] Schiano-Lomoriello D, Bono V, Abicca I, Savini G. Repeatability of anterior segment measurements by optical coherence tomography combined with Placido disk corneal topography in eyes with keratoconus. Sci Rep. 2020;10(1):1124. 31980662 10.1038/s41598-020-57926-7 PMC6981210

[46] Savini G, Schiano-Lomoriello D, Hoffer KJ. Repeatability of automatic measurements by a new anterior segment optical coherence tomographer combined with Placido topography and agreement with 2 Scheimpflug cameras. J Cataract Refract Surg. 2018;44(4):471–8. 29705008 10.1016/j.jcrs.2018.02.015

[47] Seiler TG, Mueller M, Mendes Baiao T. Repeatability and comparison of corneal tomography in mild to severe keratoconus between the anterior segment OCT MS-39 and Pentacam HR. J Refract Surg. 2022;38(4):250–5. 35412926 10.3928/1081597X-20220114-02

[48] Lei CS, Lin X, Ning R, Yu J, Huang X, Li K, et al. Repeatability and interobserver reproducibility of a swept-source optical coherence tomography for measurements of anterior, posterior, and total corneal power. Ophthalmol Ther. 2023;12(6):3263–79. 37787889 10.1007/s40123-023-00815-9 PMC10640522

