# Supplementary material for: Corneal epithelial aberrations: a novel diagnostic tool for keratoconus and forme fruste keratoconus
Source: Eye Vis (Lond). 2025 Aug 6;12:31. doi: 10.1186/s40662-025-00449-x (PMC12326862; doi:10.1186/s40662-025-00449-x)
Supplement: Supplementary file 1 — Supplementary material 1. [file 40662_2025_449_MOESM1_ESM.docx]

**Supplemental Table 1. Diagnostic performance of total corneal aberrations to differentiate FFKC from normal eyes and KC from normal eyes.**

| **Parameters** | **FFKC** | | **Mild KC** | | **Moderate KC** | | **Severe KC** | | **KC** | |
| --- | --- | --- | --- | --- | --- | --- | --- | --- | --- | --- |
|  | AUC | *P* | AUC | *P* | AUC | *P* | AUC | *P* | AUC | *P* |
| Total RMS | 0.544 | 0.343 | 0.955 | <0.001 | 0.886 | <0.001 | 0.969 | <0.001 | 1.000 | <0.001 |
| HOAs RMS | 0.701 | <0.001 | 0.988 | <0.001 | 0.957 | <0.001 | 1.000 | <0.001 | 1.000 | <0.001 |
| Coma | 0.728 | <0.001 | 0.988 | <0.001 | 0.958 | <0.001 | 0.999 | <0.001 | 1.000 | <0.001 |
| Trefoil | 0.680 | <0.001 | 0.951 | <0.001 | 0.897 | <0.001 | 0.967 | <0.001 | 0.979 | <0.001 |
| Spherical aberration | 0.625 | 0.006 | 0.621 | 0.0032 | 0.573 | 0.191 | 0.556 | 0.2673 | 0.866 | <0.001 |
| Secondary astigmatism | 0.685 | <0.001 | 0.952 | <0.001 | 0.907 | <0.001 | 0.949 | <0.001 | 0.995 | <0.001 |

AUC = area under the receiver operating characteristic curve; FFKC = forme fruste keratoconus group; KC = keratoconus group; RMS = root mean square; HOAs = higher-order aberrations

**Supplemental Table 2. Diagnostic performance of epithelial thickness to differentiate FFKC from normal eyes and KC from normal eyes.**

| **Parameters** | **FFKC** | | **Mild KC** | | | **Moderate KC** | | | **Severe KC** | | | **KC** | | |
| --- | --- | --- | --- | --- | --- | --- | --- | --- | --- | --- | --- | --- | --- | --- |
|  | AUC | *P* | AUC | *P* | AUC | | *P* | AUC | | *P* | AUC | | *P* |  |
| CET | 0.602 | 0.026 | 0.817 | <0.0001 | 0.714 | | <0.001 | 0.842 | | <0.001 | 0.875 | | <0.001 |  |
| S_1-2_ | 0.582 | 0.074 | 0.544 | 0.284 | 0.600 | | 0.074 | 0.548 | | 0.344 | 0.509 | | 0.866 |  |
| S_2-3_ | 0.578 | 0.087 | 0.621 | 0.003 | 0.528 | | 0.619 | 0.593 | | 0.067 | 0.730 | | <0.001 |  |
| S_3-4_ | 0.532 | 0.509 | 0.612 | 0.009 | 0.573 | | 0.229 | 0.593 | | 0.080 | 0.663 | | 0.003 |  |
| ST_1-2_ | 0.592 | 0.045 | 0.601 | 0.014 | 0.506 | | 0.922 | 0.584 | | 0.098 | 0.702 | | <0.001 |  |
| ST_2-3_ | 0.618 | 0.010 | 0.545 | 0.274 | 0.531 | | 0.578 | 0.523 | | 0.648 | 0.581 | | <0.001 |  |
| ST_3-4_ | 0.561 | 0.181 | 0.541 | 0.317 | 0.503 | | 0.953 | 0.504 | | 0.935 | 0.631 | | 0.014 |  |
| T_1-2_ | 0.645 | 0.002 | 0.808 | <0.001 | 0.732 | | <0.001 | 0.837 | | <0.0001 | 0.840 | | 0.664 |  |
| T_2-3_ | 0.630 | 0.005 | 0.645 | <0.001 | 0.581 | | 0.146 | 0.675 | | 0.001 | 0.608 | | 0.042 |  |
| T_3-4_ | 0.622 | 0.008 | 0.516 | 0.704 | 0.586 | | 0.125 | 0.570 | | 0.168 | 0.885 | | 0.786 |  |
| IT_1-2_ | 0.717 | <0.001 | 0.894 | <0.001 | 0.851 | | <0.001 | 0.934 | | <0.0001 | 0.503 | | 0.956 |  |
| IT_2-3_ | 0.675 | <0.001 | 0.775 | <0.001 | 0.717 | | <0.001 | 0.810 | | <0.001 | 0.888 | | <0.001 |  |
| IT_3-4_ | 0.621 | 0.008 | 0.593 | 0.022 | 0.630 | | 0.020 | 0.649 | | 0.003 | 0.730 | | <0.001 |  |
| I_1-2_ | 0.684 | <0.001 | 0.862 | <0.001 | 0.770 | | <0.001 | 0.906 | | <0.001 | 0.649 | | <0.001 |  |
| I_2-3_ | 0.643 | 0.002 | 0.659 | <0.001 | 0.546 | | 0.407 | 0.681 | | <0.001 | 0.744 | | <0.001 |  |
| I_3-4_ | 0.593 | 0.042 | 0.640 | 0.001 | 0.571 | | 0.202 | 0.685 | | <0.001 | 0.698 | | <0.001 |  |
| IN_1-2_ | 0.602 | 0.026 | 0.692 | <0.001 | 0.589 | | 0.111 | 0.723 | | <0.001 | 0.735 | | <0.001 |  |
| IN_2-3_ | 0.594 | 0.040 | 0.511 | 0.784 | 0.548 | | 0.388 | 0.544 | | 0.390 | 0.543 | | 0.414 |  |
| IN_3-4_ | 0.608 | 0.019 | 0.580 | 0.049 | 0.516 | | 0.768 | 0.666 | | 0.001 | 0.536 | | 0.503 |  |
| N_1-2_ | 0.543 | 0.345 | 0.527 | 0.509 | 0.579 | | 0.158 | 0.522 | | 0.663 | 0.512 | | 0.826 |  |
| N_2-3_ | 0.546 | 0.311 | 0.634 | 0.001 | 0.558 | | 0.301 | 0.622 | | 0.016 | 0.714 | | <0.001 |  |
| N_3-4_ | 0.580 | 0.079 | 0.524 | 0.554 | 0.520 | | 0.719 | 0.524 | | 0.635 | 0.584 | | 0.114 |  |
| SN_1-2_ | 0.556 | 0.224 | 0.593 | 0.022 | 0.600 | | 0.073 | 0.600 | | 0.050 | 0.580 | | 0.131 |  |
| SN_2-3_ | 0.563 | 0.171 | 0.647 | 0.000 | 0.552 | | 0.351 | 0.612 | | 0.028 | 0.771 | | <0.001 |  |
| SN_3-4_ | 0.560 | 0.190 | 0.544 | 0.285 | 0.519 | | 0.746 | 0.516 | | 0.747 | 0.595 | | 0.074 |  |

AUC = area under the receiver operating characteristic curve; FFKC = forme fruste keratoconus group; KC = keratoconus group; CET = central epithelium thickness; S_1–2_, ST_1–2_, T_1–2_, IT_1–2_, I_1–2_, IN_1–2_, N_1–2_, SN_1–2_ = regions located 1–2 mm from the corneal apex in the superior, superior-temporal, temporal, inferior-temporal, inferior, inferior-nasal, nasal, and superior-nasal directions, respectively; S_2–3_ to SN_2–3_ and S_3–4_ to SN_3–4_ = corresponding regions at 2–3 mm and 3–4 mm radial distances.

| **Supplemental Table 3. Diagnostic performance of stromal thickness to differentiate FFKC from normal eyes and KC from normal eyes.** | | | | | | | | | | |  |
| --- | --- | --- | --- | --- | --- | --- | --- | --- | --- | --- | --- |
| **Parameters** | **FFKC** | | **Mild KC** | | **Moderate KC** | | **Severe KC** | | **KC** | |  |
|  | AUC | *P* | AUC | *P* | AUC | *P* | AUC | *P* | AUC | *P* |  |
| CST | 0.721 | <0.001 | 0.885 | <0.001 | 0.803 | <0.001 | 0.881 | <0.001 | 0.960 | <0.001 |  |
| S_1-2_ | 0.704 | <0.001 | 0.780 | <0.001 | 0.708 | <0.001 | 0.791 | <0.001 | 0.831 | <0.001 |  |
| S_2-3_ | 0.700 | <0.001 | 0.769 | <0.001 | 0.709 | <0.001 | 0.776 | <0.001 | 0.810 | <0.001 |  |
| S_3-4_ | 0.684 | <0.001 | 0.754 | <0.001 | 0.719 | <0.001 | 0.742 | <0.001 | 0.792 | <0.001 |  |
| ST_1-2_ | 0.701 | <0.001 | 0.806 | <0.001 | 0.723 | <0.001 | 0.805 | <0.001 | 0.879 | <0.001 |  |
| ST_2-3_ | 0.693 | <0.001 | 0.764 | <0.001 | 0.702 | <0.001 | 0.767 | <0.001 | 0.814 | <0.001 |  |
| ST_3-4_ | 0.668 | 0.0002 | 0.751 | <0.001 | 0.700 | <0.001 | 0.755 | <0.001 | 0.791 | <0.001 |  |
| T_1-2_ | 0.734 | <0.001 | 0.865 | <0.001 | 0.765 | <0.001 | 0.867 | <0.001 | 0.947 | <0.001 |  |
| T_2-3_ | 0.714 | <0.001 | 0.801 | <0.001 | 0.738 | <0.001 | 0.803 | <0.001 | 0.853 | <0.001 |  |
| T_3-4_ | 0.702 | <0.001 | 0.743 | <0.001 | 0.720 | <0.001 | 0.755 | <0.001 | 0.747 | <0.001 |  |
| IT_1-2_ | 0.755 | <0.001 | 0.889 | <0.001 | 0.790 | <0.001 | 0.896 | <0.001 | 0.965 | <0.001 |  |
| IT_2-3_ | 0.748 | <0.001 | 0.801 | <0.001 | 0.753 | <0.001 | 0.813 | <0.001 | 0.829 | <0.001 |  |
| IT_3-4_ | 0.719 | <0.001 | 0.696 | <0.001 | 0.708 | <0.001 | 0.723 | <0.001 | 0.656 | <0.001 |  |
| I_1-2_ | 0.751 | <0.001 | 0.874 | <0.001 | 0.792 | <0.001 | 0.885 | <0.001 | 0.932 | <0.001 |  |
| I_2-3_ | 0.720 | <0.001 | 0.742 | <0.001 | 0.712 | <0.001 | 0.748 | <0.001 | 0.762 | <0.001 |  |
| I_3-4_ | 0.682 | <0.001 | 0.632 | 0.001 | 0.663 | <0.001 | 0.638 | 0.007 | 0.599 | 0.065 |  |
| IN_1-2_ | 0.722 | <0.001 | 0.839 | <0.001 | 0.770 | <0.001 | 0.842 | <0.001 | 0.894 | <0.001 |  |
| IN_2-3_ | 0.683 | <0.001 | 0.747 | <0.001 | 0.704 | <0.001 | 0.739 | <0.001 | 0.793 | <0.001 |  |
| IN_3-4_ | 0.660 | 0.001 | 0.663 | <0.001 | 0.661 | 0.004 | 0.656 | <0.001 | 0.672 | <0.001 |  |
| N_1-2_ | 0.708 | <0.001 | 0.820 | <0.001 | 0.751 | <0.001 | 0.811 | <0.001 | 0.890 | <0.001 |  |
| N_2-3_ | 0.691 | <0.001 | 0.772 | <0.001 | 0.717 | <0.001 | 0.762 | <0.001 | 0.830 | <0.001 |  |
| N_3-4_ | 0.678 | <0.001 | 0.759 | <0.001 | 0.718 | <0.001 | 0.752 | <0.001 | 0.803 | <0.001 |  |
| SN_1-2_ | 0.695 | <0.001 | 0.782 | <0.001 | 0.717 | <0.001 | 0.786 | <0.001 | 0.834 | <0.001 |  |
| SN_2-3_ | 0.678 | <0.001 | 0.755 | <0.001 | 0.702 | <0.001 | 0.765 | <0.001 | 0.788 | <0.001 |  |
| SN_3-4_ | 0.671 | <0.001 | 0.758 | <0.001 | 0.718 | <0.001 | 0.745 | <0.001 | 0.803 | <0.001 |  |
| AUC = area under the receiver operating characteristic curve; FFKC = forme fruste keratoconus group; KC = keratoconus group; CST = central stromal thickness; S_1–2_, ST_1–2_, T_1–2_, IT_1–2_, I_1–2_, IN_1–2_, N_1–2_, SN_1–2_ = regions located 1–2 mm from the corneal apex in the superior, superior-temporal, temporal, inferior-temporal, inferior, inferior-nasal, nasal, and superior-nasal directions, respectively; S_2–3_ to SN_2–3_ and S_3–4_ to SN_3–4_ = corresponding regions at 2–3 mm and 3–4 mm radial distances. | | | | | | | | | | |  |
|  |  |  |  |  |  |  |  |  |  |  |  |
|  |  |  |  |  |  |  |  |  |  |  |  |
|  |  |  |  |  |  |  |  |  |  |  |  |
